# Physiological responses of *Saccharomyces cerevisiae* to industrially relevant conditions: Slow growth, low pH, and high CO_2_ levels

**DOI:** 10.1002/bit.27210

**Published:** 2020-01-22

**Authors:** Xavier Hakkaart, Yaya Liu, Mandy Hulst, Anissa el Masoudi, Eveline Peuscher, Jack Pronk, Walter van Gulik, Pascale Daran‐Lapujade

**Affiliations:** ^1^ Department of Biotechnology Delft University of Technology, van der Maasweg Delft The Netherlands

**Keywords:** acid stress, carbon dioxide, carboxylic acid, yeast, zero‐growth

## Abstract

Engineered strains of *Saccharomyces cerevisiae* are used for industrial production of succinic acid. Optimal process conditions for dicarboxylic‐acid yield and recovery include slow growth, low pH, and high CO_2_. To quantify and understand how these process parameters affect yeast physiology, this study investigates individual and combined impacts of low pH (3.0) and high CO_2_ (50%) on slow‐growing chemostat and retentostat cultures of the reference strain *S. cerevisiae* CEN.PK113‐7D. Combined exposure to low pH and high CO_2_ led to increased maintenance‐energy requirements and death rates in aerobic, glucose‐limited cultures. Further experiments showed that these effects were predominantly caused by low pH. Growth under ammonium‐limited, energy‐excess conditions did not aggravate or ameliorate these adverse impacts. Despite the absence of a synergistic effect of low pH and high CO_2_ on physiology, high CO_2_ strongly affected genome‐wide transcriptional responses to low pH. Interference of high CO_2_ with low‐pH signaling is consistent with low‐pH and high‐CO_2_ signals being relayed via common (MAPK) signaling pathways, notably the cell wall integrity, high‐osmolarity glycerol, and calcineurin pathways. This study highlights the need to further increase robustness of cell factories to low pH for carboxylic‐acid production, even in organisms that are already applied at industrial scale.

## Graphical abstract

This study dissects adverse physiological effects of low pH, high CO_2_, and slow growth on *S. cerevisiae* and highlights the need to further increase robustness of cell factories to low pH for carboxylic‐acid production.

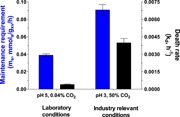

## 1 INTRODUCTION

Dicarboxylic acids are attractive platform molecules for production of a wide range of chemicals (Becker, Lange, Fabarius, & Wittmann, 2015). High‐yield microbial conversion of glucose to dicarboxylic acids can be achieved through the reductive branch of the TCA cycle and requires elevated concentrations of dissolved carbon dioxide (CO_2_) to promote carboxylation of pyruvate or phosphoenolpyruvate to oxaloacetate (Ahn, Jang, & Lee, 2016; Yin et al., 2015; Zelle, de Hulster, Kloezen, Pronk, & van Maris, 2010). Cost efficiency and sustainability of industrial dicarboxylic‐acid production can be increased by using culture pH values well below pKa_1_ of the product (pKa_1_ values of succinic, malic and fumaric acid are 4.16, 3.51, and 3.03, respectively). Production of the free acid prevents the need for coproduction of large quantities of gypsum (Abbott, Zelle, Pronk, & Van Maris, 2009; Chen & Nielsen, 2016). In contrast to most carboxylic‐acid producing prokaryotes, the yeast *Saccharomyces cerevisiae* can withstand both high CO_2_ (Aguilera, Petit, De Winde, & Pronk, 2005; Eigenstetter & Takors, 2017; Richard, Guillouet, & Uribelarrea, 2014) and low pH (Della‐Bianca, de Hulster, Pronk, van Maris, & Gombert, 2014; Verduyn, Postma, Scheffers, & van Dijken, 1990). However, although *S. cerevisiae* grows at high CO_2_, reduced biomass yields have been reported for respiring *S. cerevisiae* cultures grown at CO_2_ values of 50% and 79% (Aguilera et al., 2005; Eigenstetter & Takors, 2017; Richard et al., 2014). Similarly, *S. cerevisiae* can grow at pH values as low as pH 2.5, but only at significantly reduced specific growth rates (Carmelo, Bogaerts, & Sá‐Correia, 1996; Della‐Bianca & Gombert, 2013; Della‐Bianca, de Hulster, Pronk, van Maris, & Gombert, 2014; Eraso & Gancedo, 1987; Orij, Postmus, Beek, Brul, & Smits, 2009).

Heterotrophic microorganisms dissimilate their carbon and energy substrate to supply ATP for biomass formation and for cellular maintenance (Pirt, 1965, 1982). In yeast strains engineered for dicarboxylic‐acid production, product formation and export costs ATP and therefore directly competes with growth and maintenance processes for ATP supply (Abbott et al., 2009; Jansen & van Gulik, 2014; Maris, Konings, Dijken, & Pronk, 2004). Slow growth in fed‐batch cultures (typically at specific growth rates below 0.05 hr^−1^) limits consumption of substrate for biomass formation, which benefits product yields. However, a trade‐off of this strategy is that the fraction of the energy substrate allocated to cellular maintenance increases with decreasing specific growth rate, thereby leaving less substrate available for energy‐dependent product formation (Hensing, Rouwenhorst, Heijnen, van Dijken, & Pronk, 1995; Maurer, Kühleitner, Gasser, & Mattanovich, 2006; Wahl, Bernal Martinez, Zhao, van Gulik, & Jansen, 2017). Despite its industrial relevance, quantitative understanding of maintenance‐related processes in *S. cerevisiae* and their sensitivity to industrially relevant process conditions is far from complete. Previous studies showed that, while growth‐rate independent (Boender, de Hulster, van Maris, Daran‐Lapujade, & Pronk, 2009; Vos et al., 2016), the maintenance‐energy requirement (*m*_S_; mmol glucose/g biomass/h) of *S. cerevisiae* can be affected by the cultivation conditions (Lahtvee, Kumar, Hallstrom, & Nielsen, 2016; Liu, el Bouhaddani, Pronk, & van Gulik, 2019; Vos et al., 2016). For example, growth at pH 2.5 substantially reduces the maximum specific growth rate in batch cultures (Carmelo et al., 1996; Della‐Bianca & Gombert, 2013; Della‐Bianca et al., 2014; Orij et al., 2009) and increases activity of the plasma‐membrane proton pumps, suggesting that low pH also affects *m*_S_ (Carmelo et al., 1996; Eraso & Gancedo, 1987). Moreover, even under mildly acidic conditions, the presence of weak, membrane‐permeable organic acids strongly increases energy‐requirements for intracellular pH homeostasis (Abbott et al., 2007; Verduyn et al., 1990).

Although elevated CO_2_ and low pH are relevant industrial process conditions for dicarboxylic‐acid production and have both been reported to adversely affect yeast physiology, their effects on maintenance‐energy requirements and viability of slow growing *S. cerevisiae* cultures have not yet been quantitatively analyzed. To address this knowledge gap, a nonproducing *S. cerevisiae* laboratory strain was grown at low and near‐zero specific growth rates using a combination of glucose‐limited chemostat and retentostat cultures, at a low pH (pH 3) and elevated CO_2_ concentrations (50% CO_2_). Additionally, cultures were grown under ammonium‐limited, energy‐excess conditions at low pH. Quantitative analysis of rates, yields, and culture viability was used to dissect physiological impacts of low pH and high CO_2_. Furthermore, transcriptome analysis was employed to elucidate regulatory responses to these conditions.

## 2 MATERIALS AND METHODS

### 2.1 Strain and strain maintenance

*S. cerevisiae* CEN.PK113‐7D (Entian & Kötter, 2007; Nijkamp et al., 2012) was used in this study. The strain was stored at −80°C in 1 ml aliquots in YPD (10 g/L Bacto yeast extract, 20 g/L Bacto peptone, 20 g/L glucose) supplemented with 30% (vol/vol) glycerol.

### 2.2 Aerobic, glucose‐limited bioreactor cultures

Glucose‐limited chemostat and retentostat cultures were grown in 2‐L bioreactors (Applikon, Delft, The Netherlands) at a working volume of 1.4 L, essentially as described by Vos et al. (2016). Chemically defined medium containing 20 g/L glucose was used for chemostat and retentostat cultures. The inflowing gas (0.5 vvm) was either compressed air (0.04% CO_2_) or an in‐line mix of 50% compressed air and 50% pure CO_2_ (>99.7% purity, Linde Gas Benelux, Schiedam, The Netherlands). The two gas flows were precisely controlled with mass flow controllers (Brooks, Hatfield, PA) and mixed in a ratio of 1:1. A detailed description of preculture preparation, bioreactor operation, and medium composition is given in Supporting Information Appendix 1.

Chemostat cultures were assumed to be in steady state when, after at least five volume changes under the same process conditions, culture dry weight (see below) changed by less than 4% over two consecutive volume changes. Glucose‐limited cultures grown at pH 3 showed oscillations of CO_2_ and O_2_ concentrations in the off‐gas with a frequency of 5–8 hr, but were sampled regardless of the oscillations. These oscillations subsided upon approaching severe calorie restriction in the retentostat phase after 3 days.

### 2.3 Aerobic, ammonium‐limited bioreactor cultures

Ammonium‐limited retentostats grown at pH 3 were preceded by a chemostat phase under the same nutrient limitation, essentially as described before (Liu et al., 2019). Details on bioreactor operation and media composition of these nitrogen‐limited cultures are given in Supporting Information Appendix 1. Ammonium‐limited chemostat cultures were assumed to be in steady state when, after four volume changes, biomass dry weight, CO_2_ production rate and residual glucose and ethanol concentrations in the effluent differed by less than 5% over three consecutive volume changes.

### 2.4 Off‐gas analysis, biomass, and extracellular metabolite determinations

Concentrations of O_2_ and CO_2_ in the exhaust gas of bioreactors were quantified with a paramagnetic/infrared off‐gas analyzer (NGA 2000, Baar, Switzerland). For glucose‐limited cultures, biomass concentrations were determined by filtering duplicate, exact volumes of culture broth, diluted to an approximate concentration of 2.5 g biomass/L, over predried Supor 47 membrane filters with a 0.45 μm pore size (Pall Laboratory, Port Washington, NY) as described by Postma, Verduyn, Scheffers, and Van Dijken (1989). Biomass concentrations in ammonium‐limited cultures were analyzed by essentially the same procedure with the exception that filters were dried in an oven instead of in a microwave. Procedures for analysis of extracellular metabolites are described in detail in Supporting Information Appendix 1.

### 2.5 Viability

Viability measurements in retentostats were based on colony‐forming unit (CFU) counts, which indicate reproductive capacity of single cells (Vos et al., 2016). For glucose‐limited chemostats, CFU counts were obtained by sorting 96 single events detected by a FACS Aria™ II SORP Cell Sorter (BD Biosciences, Franklin Lakes, NJ) on a YPD plate (in quintuplicate, see Supporting Information Appendix 1 for details). To measure viability based on membrane integrity, cells were stained with the fluorescent dye propidium iodide (PI; Vos et al., 2016). Staining of single‐cell esterase activity with 5‐CFDA‐AM was used to evaluate metabolic activity (Bisschops et al., 2015). Flow cytometry was done on a BD‐Accuri C6 with a 488 nm excitation laser (Becton Dickinson, Franklin Lakes, NJ). For each sample, over 10,000 events in fluorescence channel 3 (670 LP) were analyzed for PI and in fluorescence channel 1 (530/30 nm) for 5‐CFDA‐AM. The forward‐scatter height (FSC‐H) threshold was set to 80,000.

### 2.6 Regression analysis of biomass accumulation in glucose‐limited retentostats

Quantification of maintenance‐energy requirements and death rate in glucose‐limited retentostats was done by model‐based regression analysis of biomass accumulation over time (Vos et al., 2016). The fitted model parameters were a constant first‐order death rate and a growth‐rate independent maintenance‐energy coefficient. The maximum theoretical yield of biomass on substrate (*Y*_x/s_ max) was set to a fixed value of 0.5 g_X_/g_S_. This analysis generated quantitative estimates of specific growth rate and glucose consumption rates during the first, dynamic phase of retentostat cultivation (see Section 3).

### 2.7 Carbon and nitrogen balances and rate calculations

Carbon and nitrogen recoveries were calculated based on measurements of substrate and product concentrations in the gas and liquid phases and gas and liquid in‐ and outflow rates. Ethanol evaporation from bioreactors was quantified (Cueto‐Rojas, Seifar, Pierick, Heijnen, & Wahl, 2016) and taken into account in the calculation of specific ethanol‐production rates. Specific growth rates in nitrogen‐limited retentostat cultures were calculated as described by (Boender et al., 2009).

### 2.8 Transcriptome analysis

Detailed descriptions of sampling procedures (Mendes et al., 2013; Piper et al., 2002) total RNA extraction (Schmitt, Brown, & Trumpower, 1990), mRNA enrichment and RNA sequencing (Novogene, Hong Kong, China & Baseclear, Leiden, The Netherlands), alignment (STAR; Dobin et al., 2013) and mapping (ht‐seq count; Anders, Pyl, & Huber, 2015) of reads against the S288C genome (Engel et al., 2014), TMM‐normalization (EdgeR R‐package; Robinson, McCarthy, & Smyth, 2009), gene set enrichment (piano R‐package; Väremo, Nielsen, & Nookaew, 2013) and trend analysis with the regression‐based growth rate (see above) as variable (maSigPro R‐package; Conesa, Nueda, Ferrer, & Talon, 2006; Nueda, Tarazona, & Conesa, 2014) are provided in Supporting Information Appendix 1. Transcriptome data are available are Gene Omnibus (https://www.ncbi.nlm.nih.gov/geo/) under accession number GSE133136.

### 2.9 Biomass composition, glycogen, and trehalose determination

Biomass elemental composition and biomass protein content were quantified as described previously (Lameiras, Heijnen, & van Gulik, 2015; Lange & Heijnen, 2001). After sampling for analysis of the intracellular storage carbohydrates glycogen and trehalose (Vos et al., 2016), pellets were stored at −80°C. Samples were processed (Parrou & François, 1997) and analyzed as described in Supporting Information Appendix 1.

### 2.10 Metabolic flux analysis (MFA)

MFA was performed as described previously (Daran‐Lapujade et al., 2004) with two modifications to the stoichiometric model: biomass composition was re‐defined based on measured biomass elemental composition and reduction of acetaldehyde to ethanol was incorporated as ethanol was a main product of ammonium‐limited aerobic cultures.

## 3 RESULTS

### 3.1 Low pH and high CO_2_ levels cause increased death rate and maintenance‐energy requirements in glucose‐limited retentostat cultures of *S. cerevisiae*

**Figure 1:**
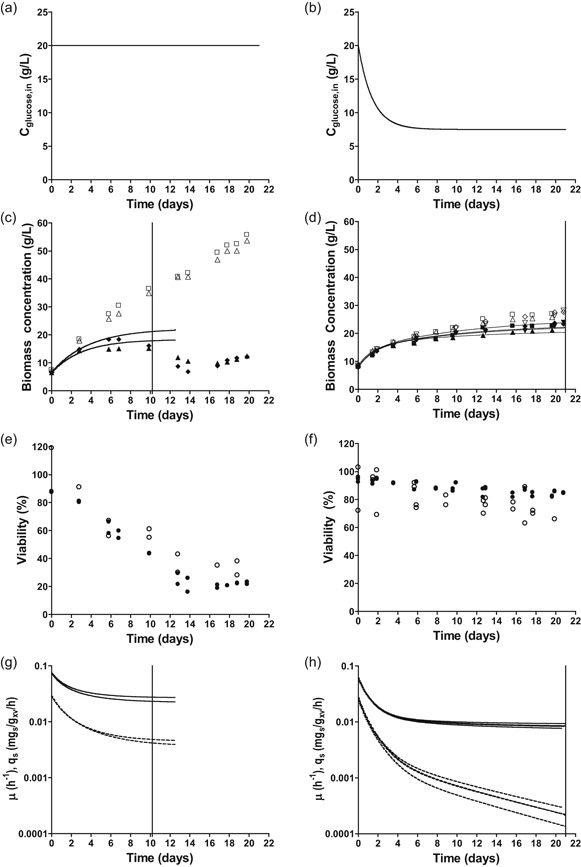
Physiological characterization of *S. cerevisiae* CEN.PK113‐7D in duplicate glucose‐limited, aerobic retentostat cultures, grown at pH 3 and 50% CO_2_ (left column) and in quadruplicate cultures grown under reference conditions (pH 5, 0.04% CO_2_; Vos et al., 2016). (a, b) Glucose concentration in influent during retentostat cultivation. (c, d) Biomass dry weight (open symbols) and viable biomass dry weight estimated by PI staining (closed symbols). The vertical line indicates the time until which data points were included in regression analysis for biomass accumulation (see main text for detailed explanation). (e, f) Viability of retentostat cultures based on PI staining (closed symbols) and CFU (open symbols). (g, h) Regression‐based biomass‐specific growth rate (μ, dashed lines) and biomass‐specific glucose uptake rate (q_s_ plain lines) during the first 10 days of retentostat cultivation. Viable biomass concentrations used for regression analysis were based on PI staining. The vertical line indicates the time until which data points were included in the regression analysis for biomass accumulation (see main text for detailed explanation). CFU, colony‐forming unit; PI, propidium iodide

The physiological responses of the *S. cerevisiae* laboratory strain CEN.PK113‐7D under conditions relevant for industrial dicarboxylic acid production (aerobic, 50% CO_2_, pH 3.0) were investigated at near‐zero growth rates in retentostat cultures. In these retentostat cultures, a filter in the effluent line enabled full biomass retention (Ercan et al., 2015). At a constant feed rate of glucose, biomass accumulates and the supplied substrate per cell gradually decreases and growth ceases until virtually all substrate is used to fulfill maintenance‐energy requirements (Boender et al., 2009; Vos et al., 2016). Because the industrially relevant conditions applied in this study were expected to increase m_S_ relative to standard laboratory conditions (i.e., pH 5.0 and sparging with air; Lahtvee et al., 2016; Vos et al., 2016), the asymptotic decrease of the glucose concentration in the feed, as previously applied for laboratory conditions (Vos et al., 2016), was not applied (Figures 1a and 1b). Instead, the substrate concentration in the feed was kept constant. This higher rate of substrate supply enabled the culture dry weight to accumulate to higher concentrations (Figures 1c and 1d). Culture viability, based on membrane integrity (PI staining) and reproductive capacity (CFU) was substantially lower under the industrially relevant conditions than under standard laboratory conditions (Figures 1e and 1f). Furthermore, the lower viable biomass concentration at near‐zero growth rates in the retentostat cultures grown under industrially relevant conditions indicated a higher *m*_S_ than under laboratory conditions.

Time‐dependent regression analysis of substrate and product concentrations was previously shown to enable accurate estimates of specific growth rate, specific substrate‐consumption rate, first‐order death rate and m_S_ in carbon‐ and energy‐limited yeast retentostat cultures (Vos et al., 2016). In contrast to growth under standard laboratory conditions, growth under industrially relevant conditions caused a strong decrease of the viable biomass concentration after the first 10 days of cultivation, which prevented use of regression analysis for data obtained beyond Day 10 (Figures 1c and 1g).

**Figure 2:**
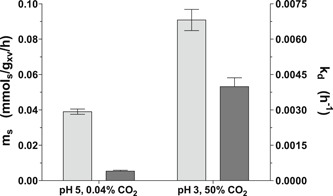
Maintenance‐energy requirements and first‐order death rate in pH 5, 0.04% CO_2_ reference conditions and in industrially relevant pH 3, 50% CO_2_ conditions in carbon‐limited retentostat cultures of *S. cerevisiae* CEN.PK113‐7D. These parameters were derived based on regression analysis of the biomass and viable biomass accumulation (see Section 2 and Supporting Information Appendix 1 for details). Light gray bars and dark gray bars present maintenance energy requirements and first‐order death rates, respectively

Regression analysis showed that, although higher than the lowest growth rate reached under laboratory conditions (0.0008 hr^−1^, Figure 1h), the specific growth rate of retentostat cultures grown under the industrially relevant conditions was already extremely low at 10 days of cultivation (0.0045 ± 0.0003 hr^−1^, Figure 1g). This difference was partially due to an 8‐fold higher death rate under industrially relevant conditions than under laboratory conditions (0.0039 ± 0.0005 hr^−1^ vs 0.00047 hr^−1^; Figure 2). Moreover, the m_S_ calculated by regression analysis was more than 2‐fold higher under industrially relevant conditions (0.0908 ± 0.0085 mmol_s_/g_x_ viable biomass/h vs 0.039 ± 0.003 mmol_s_/g_x_ viable biomass/h, Figure 2). Throughout retentostat cultivation, residual glucose concentrations remained between 0.01 and 0.07 mM. These results demonstrate that the combination of an extremely low growth rate, low pH and high CO_2_ has marked adverse effects on the physiology of *S. cerevisiae*.

### 3.2 High maintenance‐energy requirements and death rates result from low pH rather than high CO_2_ levels

To further explore the extreme physiological response of *S. cerevisiae* in retentostat cultures grown under industrially relevant conditions, the effects of low pH and high CO_2_ concentration at low growth rates were investigated separately and in combination. These experiments were performed in glucose‐limited chemostat cultures grown at the same dilution rate (0.025 hr^−1^) as the retentostats, but without cell retention (Figure 1). In energy‐limited chemostat cultures grown at a fixed dilution rate, differences in biomass yield (Y_x/s_) can provide strong indications for differences in maintenance‐energy requirements (Lahtvee et al., 2016). Under laboratory conditions (low CO_2_, pH 5) at 0.025 hr^−1^, *S. cerevisiae* invests ca. 20% of the consumed glucose in cellular maintenance (Vos et al., 2016), resulting in a biomass yield of 0.416 ± 0.005 g_x_/g_s_. Despite small deviations in medium composition (higher concentrations of biotin and iron sulfate in the present study), the biomass yield of 0.419 ± 0.009 g_x_/g_s_ measured in the present study was entirely consistent with the yield observed by (Vos et al., 2016).

Irrespective of culture pH, increasing CO_2_ levels to 50% did not significantly affect biomass yields at a dilution rate of 0.025 hr^−1^ relative to those observed under standard laboratory conditions (Table 1). Conversely, growth at pH 3 led to a significantly lower biomass yield than at pH 5, both at standard and at elevated CO_2_ Levels (7.4% and 9.7% decrease, respectively; 0.419 ± 0.009 g_x_/g_s_ vs 0.388 ± 0.005 g_x_/g_s_; *p* < .001 for pH 5 vs pH 3 when sparged with compressed air and 0.411 ± 0.006 g_x_/g_s_ vs 0.371 ± 0.004 g_x_/g_s_; *p* < .02 for pH 5 vs pH 3 at 50% CO_2_). These results showed that the higher m_s_ in retentostat cultures grown at high CO_2_ and low pH resulted from the low pH rather from the high CO_2_.

**Table 1:** Physiology of *S. cerevisiae* CEN.PK113‐7D in aerobic glucose‐limited chemostat cultures grown at a dilution rate of 0.025 hr^−1^

	pH 5		pH 3	
CO_2_ in inlet gas (%)	0.04	50	0.04	50
Culture replicates	4	5	3	4
D (hr^−1^)	0.026 ± 0.001	0.025 ± 0.001	0.025 ± 0.001	0.025 ± 0.000
Biomass yield (g_x_/g_s_)	0.419 ± 0.009	0.409 ± 0.005	0.388 ± 0.005	0.372 ± 0.004
Viability PI (%)	97 ± 1	96 ± 4	71 ± 1	85 ± 3
Viability CFDA (%)	96 ± 2	98 ± 0	81 ± 2	92 ± 2
Viability CFU‐FACS (%)	92 ± 1^2^	85 ± 10^5^	73 ± 2^2^	74 ± 1^2^
*μ* (hr^−1^)	0.027 ± 0.001	0.026 ± 0.000	0.035 ± 0.001	0.030 ± 0.001
*q*_glucose_ (mmol/g_xv_/hr)	0.358 ± 0.016	0.357 ± 0.008	0.508 ± 0.011	0.443 ± 0.023
*q*_o2_ (mmol/g_xv_/hr)	1.019 ± 0.087	ND	1.361 ± 0.102	ND
*q*_co2_ (mmol/g_xv_/hr)	1.042 ± 0.094	ND	1.394 ± 0.138	ND
C_glucose_ (g/L)	0.011 ± 0.003	0.013 ± 0.001	0.005 ± 0.003	0.010 ± 0.006
Carbon recovery (%)	100.0 ± 4.1	ND	93.0 ± 4.5	ND
RQ (*q*_co2_/*q*_o2_)	1.023 ± 0.016	ND	1.024 ± 0.043	ND
Glycogen content (mg/g_x_)	35.3 ± 3.3	32.6 ± 2.2	46.4 ± 3.2^2^	30.4 ± 1.9
Trehalose content (mg/g_x_)	19.4 ± 3.7	18.3 ± 1.94	12.64 ± 1.1^2^	9.7 ± 1.7

*Note*: “Replicates” indicates the number of biological replicates. Superscripts indicate the number of biological replicates for individual analyses when these deviate from the number presented under “Replicates.” ND: not determined. Biomass specific rates (*q* values) were calculated based on viable biomass (xv), estimated by PI staining. At pH 3 the cultures showed oscillations in dissolved oxygen, exhaust CO_2_ and exhaust oxygen levels, regardless of the percentage of CO_2_ in the inlet gas.

Measurements, by three different methods (CFU, PI, and CFDA staining, Table 1 and Supporting Information Appendix 2), showed that, irrespective of CO_2_, low pH led to a strongly reduced viability of glucose‐limited chemostat cultures. Conversely, increasing the CO_2_ levels did not significantly affect culture viability. Assuming that cells measured as nonviable did not contribute to biomass formation or glucose consumption, specific rates were corrected for viability based on PI staining, resulting in higher specific growth rates (*μ*) and biomass‐specific substrate uptake rates (Table 1).

### 3.3 Growth under ammonium‐limited, energy‐excess conditions does not reduce death rates at low pH and increases nongrowth associated glucose consumption rates

**Figure 3:**
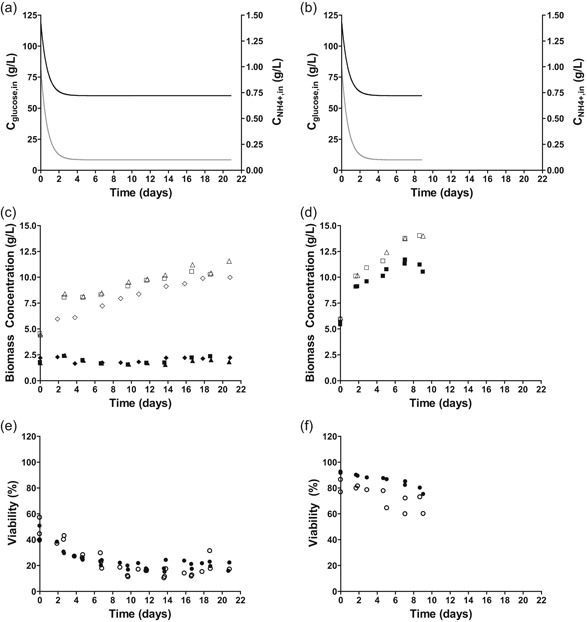
Physiological characterization of *S. cerevisiae* CEN.PK113‐7D in triplicate nitrogen‐limited retentostat cultures at pH 3 (left column) and in duplicate reference condition at pH 5 (Liu et al., 2019). (a, b) Medium glucose (black line) and nitrogen (gray line) concentration during retentostat cultivation. (c, d) Biomass accumulation for cell dry weight (open symbols) and viable biomass (closed symbols) quantified by PI staining. (e, f) Viability of retentostat cultures based on PI staining (closed symbols) and CFU (open symbols). CFU, colony‐forming unit; PI, propidium iodide

Since glucose acts as energy substrate as well as carbon source, the high death rates and maintenance‐energy requirements observed at pH 3 might reflect a cellular energy shortage. Therefore, physiological responses of *S. cerevisiae* were also investigated in near‐zero growth rate retentostat cultures grown at pH 3 and pH 5 under ammonium‐limited, glucose‐excess conditions. These cultures were started from ammonium‐limited chemostat cultures grown at a low dilution rate of 0.023 hr^−1^. The biomass concentration increased during the first 15 days of retentostat cultivation, after which it stabilized (Figure 3c). Culture viability in ammonium‐limited chemostats grown at pH 3 (50%; Figure 3e) was very low in comparison with viabilities observed in glucose‐ and ammonium‐limited chemostat cultures grown at pH 5 (Figures 1f and 3f) and in glucose‐limited cultures grown at pH 3 (Figure 1e). During ammonium‐limited retentostat cultivation at pH 3, the total viable biomass concentration did not increase significantly (Figure 3c). Based on biomass and viability measurements towards the end of the retentostat experiments, the specific growth rate had decreased to 0.0006 ± 0.0001 hr^−1^ (Table 2). As the viable biomass concentration remained virtually constant during retentostat cultivation, this growth rate equaled the death rate. The combination of nitrogen‐limited growth and its associated excess availability of glucose clearly did not prevent adverse effects of low pH at near‐zero growth rates. However, the substantially lower death rate in ammonium‐limited retentostats indicated that growth under energy‐source excess enabled *S. cerevisiae* to better survive prolonged exposure to low‐pH stress than energy‐source‐limited growth.

**Table 2:** Physiology of *S. cerevisiae* CEN.PK113‐7D in aerobic ammonium‐limited chemostat and retentostat cultures at pH 3

	Chemostat	End retentostat
*D* (hr^−1^)	0.023 ± 0.004	0.023 ± 0.004
*µ* (hr^−1^)	0.053 ± 0.001	0.0006 ± 0.0001
Yield (g_x_/g_glucose_)	0.048 ± 0.002	0.0016 ± 0.0002
Viability PI (%)	43 ± 5	20 ± 3
Viability CFDA (%)	46 ± 3	12 ± 3
Viability CFU (%)	47 ± 7	17 ± 6
*q*_glucose_ (mmol/g_xv_/hr)	6.1 ± 0.4	2.2 ± 0.2
*q*_o2_ (mmol/g_xv_/hr)	2.00 ± 0.3	0.83 ± 0.16
*q*_co2_ (mmol/g_xv_/hr)	12.5 ± 0.8	4.6 ± 0.2
*q*_ethanol_ (mmol/g_xv_/hr)	10.2 ± 0.6	4.1 ± 0.5
*q*_byproduct_ (mmol/g_xv_/hr)	0.36 ± 0.01	0.18 ± 0.06
*Y*_ethanol/glucose_ (mol/mol_s_)	1.71 ± 0.03	1.83 ± 0.09
C_glucose_ (g/L)	33.77 ± 1.09	11.22 ± 0.15
Carbon recovery (%)	99 ± 1	100 ± 2
RQ value (q_CO2_/q_O2_)	6.8 ± 1.2	5.9 ± 1.0
*q*_N,in_ (mmol_N_/g_xv_/hr)	0.079 ± 0.002	0.0034 ± 0.000
*q*_N,out_ (mmol_N_/g_xv_/hr)	0.007 ± 0.000	0.0022 ± 0.000
*q*_N,X_ (mmol_N_/g_xv_/hr)	0.073 ± 0.001	0.0014 ± 0.000
C_N_ (g/L)	BDL	BDL
Nitrogen‐recovery (%)	101.0 ± 1.0	106 ± 9
Glycogen content (mg/g_x_)	22 ± 2.0	66 ± 1.8
Trehalose content (mg/g_x_)	35 ± 0.3	20 ± 1.0
Biomass composition	C1H1.87O0.63N0.089	C1H1.85O0.59N0.061
	P0.012S0.0016	P0.012S0.0012

*Note*: Data present the average and standard deviation of triplicate experiments from steady‐state (chemostat) and near‐zero growth (retentostat) cultures. *q*'s indicate biomass specific values. Subscripts indicate the considered compound. X, biomass; byproducts, the sum of acetate, succinic acid, lactic acid and glycerol; N,in, nitrogen consumed; N,out, sum of nitrogen excreted in the form of protein and free amino acids; N,X, nitrogen conserved in biomass. BDL, below detection limit.

Throughout the ammonium‐limited retentostat cultivation, residual glucose concentrations remained above 10 g/L, confirming that cultures were not energy‐limited. Ethanol concentrations remained below 15 g/L and, therefore, below reported toxic levels (Fujita, Matsuyama, Kobayashi, & Iwahashi, 2006). Residual ammonium concentrations were below detection limit (0.02 mg/L) in all samples. In ammonium‐limited chemostat cultures 93% of the supplied nitrogen was recovered in biomass. In contrast, only 35–40% of supplied nitrogen was used for biomass formation after prolonged ammonium‐limited retentostat cultivation. The remaining 60–65% of the supplied nitrogen was lost in the effluent as proteins and peptides (Table 2).

**Figure 4:**
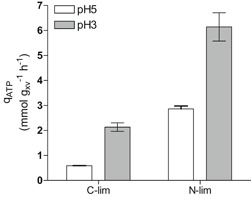
Maintenance energy requirements (glucose‐limited cultures; C‐lim) and nongrowth associated energy requirements (ammonium‐limited cultures; N‐lim) of *S. cerevisiae* CEN.PK113‐7D during growth at pH 5 and at pH 3 in retentostat cultures based on metabolic flux analysis. White bars: pH 5, gray bars: pH 3 (and 50% CO_2_ for glucose‐limited cultures). Data for glucose‐limited cultures grown at pH 5 are from Vos et al. (2016), data for ammonium‐limited cultivation at pH 5 are from Liu et al. (2019)

The nonconstant death rate of the nitrogen‐limited retentostat cultures prevented use of the regression model to estimate maintenance‐energy requirements. Instead, MFA was used to derive and compare rates of ATP turnover in the absence of growth at the end of the glucose‐ and ammonium‐limited retentostat experiments (vertical line in Figure 1c,d and final points in Figure 3c,d; input parameters used for the MFA are specified in Supporting Information Appendix 3. Because the biomass protein content was much lower in the ammonium‐limited cultures, a condition‐dependent biomass composition (Table 2) was a key input to the MFA‐model. For the glucose‐limited cultures, a previously reported biomass composition for glucose‐limited chemostat cultures of the same strain was used (D = 0.022 hr^−1^, Lange & Heijnen, 2001). Additionally, the in vivo P/O‐ratio was assumed to be 1.0 (Verduyn, Stouthamer, Scheffers, & van Dijken, 1991). The ATP hydrolysis rate derived from the MFA model for glucose‐limited cultures at pH 5 closely matched the m_ATP_ derived from the regression model (Figures 2 and 4). Under glucose limitation, a decrease in pH from 5 to 3 resulted in a 3.7 fold increase of the calculated ATP‐hydrolysis rate at near‐zero growth rates (0.58 and 2.13 mmol_ATP_/g_x_ viable/hr, respectively). The differences between the ATP‐hydrolysis rate at pH 3 derived from MFA (Figure 4) and the m_ATP_ from the regression model at pH 3 under glucose‐limitation (Figure 2, estimated with a P/O‐ratio of 1.0) can be explained by the different method of parameter estimation and the residual growth due to the high death rates under this condition. At pH 3, this nongrowth associated rates of ATP turnover was 2.9 fold higher in ammonium‐limited retentostats (6.14 mmol_ATP_/g_x_ viable/h) than in the corresponding glucose‐limited cultures (Figure 4).

### 3.4 Growth at low pH and/or high CO_2_ cause extensive transcriptional rearrangements

**Figure 5:**
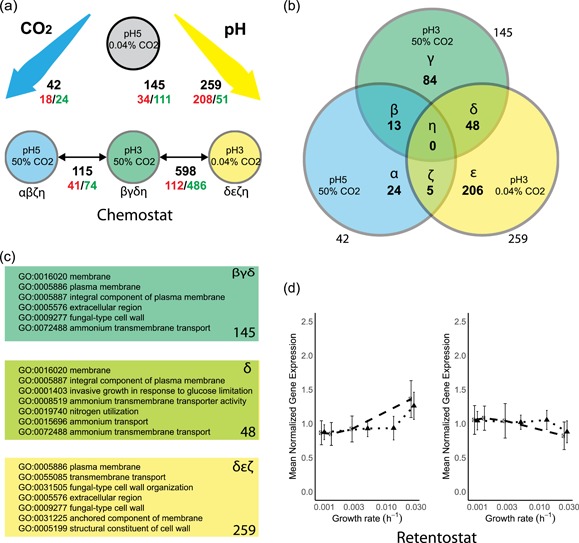
Differential gene expression and gene set analysis in response to high CO_2_, low pH, the combination of high CO_2_ and low pH (a–c) and to near‐zero growth rates (d–f). (a) Pairwise comparisons between steady‐state chemostat conditions to high CO_2_ (blue), low pH (yellow), its combination (green) versus a “laboratory conditions” reference (gray), as well as against the combination of the conditions (low pH, high CO_2_). Black numbers indicate total number of differentially expressed genes (|FC| > 2, FDR < 0.005, see Section 2), red numbers indicate upregulated genes, green numbers indicate downregulated genes. (b) Venn diagram of total DE genes based on pairwise comparison against the “laboratory conditions” reference, corresponding to the black numbers in panel a. Sections in the Venn diagram are indicated with Greek letters (α‐η). (c) Enriched Gene Ontology sets based on hyper‐geometric distribution analysis (Bonferroni corrected *p* < 0.05 for: pH 3, 50% CO_2_ (145 genes, dark green top panel, corresponding to βγδ in panel b); the overlap between pH 3 and pH 3, 50% CO_2_ conditions (48 genes, light green middle panel; δ in panel b); low pH conditions (259 genes, yellow bottom panel, δεζ in panel b)). See Supporting Information Appendix 4 for full tables. (d) Mean‐normalized gene expression for genes with a positive (left) and negative (right) correlation with specific growth rate, based on Vos et al. for “laboratory conditions” (black dots, dashed line) and “industrial conditions” (black triangles, dotted line). Error bars represent standard deviation of the mean‐normalized expression of the gene set. Bonferroni corrected *p* values for the “laboratory conditions (pH 5, 0.04% CO_2_),” up: 1.46E−89, down: 1.9E−4. “industrial conditions” (pH 3, 50% CO_2_), up: 2.96E−26, down: 9.8E−16. DE, differentially expressed; FC, fold change; FDR, false‐discovery rate [Color figure can be viewed at wileyonlinelibrary.com]

Transcriptional responses of glucose‐limited chemostat cultures to high CO_2_, low pH or both was explored to gain further insight in the mechanisms underlying the reduced biomass yield, the increased maintenance energy requirements and increased cell death under industrially relevant conditions. Pair‐wise differential gene‐expression analysis against the reference at pH 5% and 0.04% CO_2_ (absolute fold‐change (FC) > 2 and false‐discovery rate (FDR) < 0.005, see Section 2) revealed large differences in yeast transcriptional responses to the different conditions for 50% CO_2_ alone (42 genes, blue), pH 3 alone (259 genes, yellow) and 50% CO_2_ and pH 3 combined (145 genes, green) (Figure 5a, Greek letters correspond with subsets in Figure 5b).

To investigate common and specific responses to high CO_2_ and low pH conditions, the corresponding sets of differentially expressed genes were analyzed (Figure 5b, sections in Venn diagram denoted with α‐η). A set of 42 genes that were differentially expressed in response to high CO_2_ only (Figure 5b, αβζ) did not reveal a clear enrichment for specific functional categories. The largest response was observed at pH 3, with 267 differentially expressed genes (Figure 5b, δεζ). This gene set showed an overrepresentation of genes involved in plasma‐membrane and cell‐wall organization (Figure 5c, δεζ, yellow). The same functional categories were overrepresented among 154 genes that were differentially expressed (Figure 5b, βγδ) when high CO_2_ and low pH were combined (Figure 5c, βγδ, green).

A set of 13 genes that, irrespective of culture pH, were differentially expressed in response to high CO_2_ (Figure 5b, β) consisted of genes involved in gluconeogenesis (*ICL1*, *PKC1* and *FBP1*, all upregulated at high CO_2_), while *NCE103*, encoding carbonic anhydrase, was downregulated. Among 48 genes that were differentially expressed in response to low pH, both at high and low CO_2_ (Figure 5b, δ), genes involved in ammonium transport and plasma‐membrane processes were overrepresented (Figure 5c, δ). This set comprised 18 genes that were commonly upregulated, 18 that were commonly downregulated and 12 genes that displayed opposite responses to low pH at low and high CO_2_ (see Supporting Information Appendix 4). Of the latter 12 genes, five (*PIR4*/*YJL158C*, *TIP1*/*YBR067C*, SVS1/*YPL163C*, *SRL1*/*YOR247W*, *TIR2*/*YOR010C*) encode cell‐wall proteins, with *PIR4*, *TIP1*, *SRL1*, and *TIR2* described as mannoproteins. An unexpectedly large transcriptional response, involving no fewer than 598 genes (Figure 5a), was observed in response to high CO_2_ at low pH. Of this large set of genes, many encoded proteins involved in processes related to cell wall, cell membrane and ergosterol biosynthesis (Supporting Information Appendix 4).

The scale of transcriptional response to glucose limited retentostat cultivation at near‐zero growth rates was similar for laboratory and industrial conditions, with 569 and 531 differentially expressed genes, respectively (Figure 5d). Notable differences between laboratory and industrial conditions included the regulation of *PDR12*, which encodes a plasma‐membrane transporter in weak organic acid tolerance (Piper et al., 1998; Ullah, Orij, Brul, & Smits, 2012), that responded in opposite directions under the two conditions, and the enrichment of genes encoding extracellular proteins and/or involved in cell wall processes among the genes whose expression was positively correlated with increasing growth rate under laboratory conditions but not industrial conditions (Supporting Information Appendix 5).

## 4 DISCUSSION

This study was designed to quantify and dissect adverse physiological effects on *S. cerevisiae* of process conditions that are relevant for dicarboxylic acid production (low pH, high CO_2_, and slow growth). Elevated CO_2_ (50%) did not, by itself, affect the biomass yield or viability of *S. cerevisiae* as compared to those under reference conditions (Table 1), and, accordingly, triggered only a weak transcriptional response (Figure 5). This result appears to contradict results from two independent previous studies on the same strain, performed at CO_2_ levels of 50% and 79%, under fully respiratory conditions (Aguilera et al., 2005; Eigenstetter & Takors, 2017; Richard et al., 2014). This apparent discrepancy may be related to the lower specific growth rates applied in the present study (0.025 hr^−1^ and below, while the cited earlier studies used 0.10 hr^−1^). Indeed, robustness of *S. cerevisiae* to various other stresses is inversely correlated with growth rate (Bisschops et al., 2017; Boender et al., 2011; Brauer et al., 2008; Lu, Brauer, & Botstein, 2009). Additionally, in agreement with the present study, Eigenstetter and Takors observed a recovery from the CO_2_ stress after five generations.

**Figure 6:**
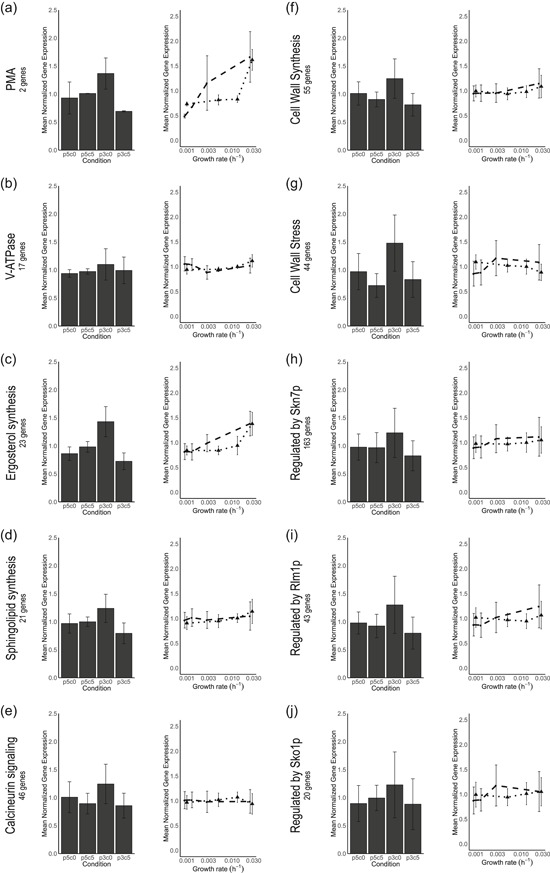
Transcriptional responses of gene sets related to proton homeostasis and diffusion (a–d), genes responsive to signaling pathways involved in low pH stress (e, h, i, j) and cell wall synthesis (f) from (Lesage & Bussey, 2006) and cell wall stress (g) from (Boorsma et al., 2004). The number of genes in each gene set is indicated in the panels. Left figures indicate per gene mean‐normalized expression from chemostats. Right figures indicate the per gene mean‐normalized expression versus growth rate for retentostat cultures under “laboratory” condition (black dots, dashed line) and “industrial” conditions (black triangles, dotted line). Mean‐normalization was performed on the separate experiments and prohibits intercomparing the expression levels. Error bars in each plot indicate the standard deviation of the per gene mean‐normalized expression of all genes in the subset

In contrast to the apparent insensitivity of slow‐growing cultures to high CO_2_, a low culture pH caused increased maintenance‐energy requirements in glucose‐limited cultures, both at high and at low CO_2_ (Figures 2 and 4). Moreover, both in glucose‐ and in ammonium‐limited cultures, growth at low pH led to a reduced culture viability. A low extracellular pH results in a large proton gradient across the cell membrane and might increase proton influx via passive diffusion. To maintain intracellular pH homeostasis, *S. cerevisiae* can expel protons via the plasma‐membrane ATPase Pma1 (Carmelo et al., 1996; Eraso & Gancedo, 1987), a process that is an intrinsic part of maintenance‐energy metabolism (Figure 4). In glucose‐limited chemostat cultures, no changes in the expression of genes encoding for proteins involved in proton homeostasis, including *PMA1* and genes encoding subunits of the vacuolar V‐ATPase, were observed. However, in glucose‐limited retentostat cultures, *PMA1* and *PMA2* expression did show a positive correlation with specific growth rate (Figures 6a and 6b).

During ammonium‐limited growth, additional mechanisms might explain the increase in nongrowth associated energy requirements (Figure 4). Futile cycling of ammonia and ammonium across the plasma membrane could require addition proton pumping via Pma1 (Cueto‐Rojas et al., 2017; Liu et al., 2019) and might be aggravated at low pH. Additionally, presence of ethanol in the ammonium‐limited cultures (up to 15 g/L) might stimulate proton leakage across the plasma membrane and thus trigger an increase in ATP‐mediated proton export (Lindahl et al., 2017; Madeira et al., 2010) Together, the results of this study indicate that high death rates of slow‐growing cultures at low pH cannot be directly attributed to energy‐limited growth or increased maintenance energy‐requirements.

Yeast transcriptional responses to near‐zero growth rates in glucose‐limited retentostat cultures were highly similar under laboratory and industrially relevant conditions, indicating that the different death rates and maintenance‐energy requirements under these conditions (Figure 1) did not trigger extensive transcriptional reprogramming. In chemostat cultures, pronounced transcriptional responses to low pH involved many genes involved in cell wall synthesis and stress. Proteins located outside the plasma membrane, including cell wall proteins, are directly exposed to the extracellular medium. As the isoelectric point (pI) of a protein determines its folding and functionality, activity of these proteins may be particularly sensitive to low extracellular pH (Schwartz, Ting, & King, 2001). Failure to replace inactive extracellular proteins, either through accumulation of inactive protein or through a limited capacity for their replacement, may therefore be a key contributor to cell death, increased maintenance energy requirements or both at low pH.

While neither synergistic nor antagonistic physiological effects of low pH and high CO_2_ were observed, transcriptional responses to the combination of these environmental conditions strongly differed from the transcriptional responses to either low pH or high CO_2_ (Figure 5a). In particular, high CO_2_ levels appeared to dampen the transcriptional response to low pH. Low pH stress triggers transcriptional regulation of genes under control of the cell wall integrity (CWI), high‐osmolarity glycerol (HOG) and calcineurin signaling pathways (de Lucena et al., 2015) and cytosolic pH acts as a sensor for PKA‐signaling (Dolz‐Edo, Guikema‐van der Deen, Brul, & Smits, 2019; Orij et al., 2012). Additionally, sensing of CO_2_ is relayed through sphingolipid‐mediated sensing, via the kinases Pkh1 and Pkh2, to the central nutrient sensor Sch9 (Pohlers et al., 2017). Extensive crosstalk between these signaling pathways enables cellular homeostasis (Chen & Thorner, 2007; Deprez, Eskes, Wilms, Ludovico, & Winderickx, 2018; Flamigni & Dolci, 2010; Fuchs & Mylonakis, 2009; Rodriguez‐Pena, Garcia, Nombela, & Arroyo, 2010). Accordingly, genes under control of the transcription factors regulated by these signaling pathways (Skn7p, Rlm1p, Sko1p, Figures 6e, 6h, 6i, and 6j) were upregulated at pH 3, as were gene sets involved in cell wall synthesis (Lesage & Bussey, 2006) and cell wall stress (Boorsma et al., 2004). However, these gene sets did not respond during growth at pH 3 at 50% CO_2_ (Figures 6f and 6g). While the present data do not enable elucidation of the precise nature of the cross‐talks between pH and CO_2_ signaling, in *S. cerevisiae*, two interactions between the abovementioned signaling pathways could provide further leads of investigation. First, CWI is sensed by the GPI‐anchored nano‐spring Wsc1 (Dupres et al., 2009), ultimately activating CWI and PKA pathways (García et al., 2017). The kinases Pkh1 and Pkh2 that relay the CO_2_ signal to Sch9 are also essential for Pkc1 activation of the CWI pathway (Inagaki et al., 1999; Levin, 2005; Pohlers et al., 2017) and phosphorylate the kinases Ypk1 and Ypk2 that in turn phosphorylate the CWI MAP Kinase Mpk1/Stl2 (Roelants, Torrance, Bezman, & Thorner, 2002; Schmelzle, Helliwell, & Hall, 2002). Second, at high extracellular CO_2_ conditions bicarbonate accumulates intracellularly, improves buffering of the cytosol, and attenuates the cytosolic pH (Buck & Levin, 2011; Eigenstetter & Takors, 2017). Both the cytosolic pH and bicarbonate are direct signals for PKA signaling (Buck & Levin, 2011; Dolz‐Edo et al., 2019; Thomas, 1976). Phosphoproteomic analysis of the proteins in the CWI, HOG, and PKA signaling pathways could prove an efficient strategy to elucidate the observed interplay of high CO_2_ and low pH signaling (Mascaraque et al., 2013), which could be supported by analysis of the in vivo cytosolic pH at high CO_2_ and low pH conditions by the pH‐dependent GFP‐derivative pHluorin (Orij et al., 2012).

The present study indicates that sensitivity to high CO_2_ is unlikely to be a major concern for the development of robust yeast cell factories for production of dicarboxylic acids. Instead, minimizing maintenance‐energy requirement and death rate at low pH was identified as a major objective for strain improvement. Even in the absence of product formation, low pH was shown to augment the trade‐off, at low specific growth rates, between a reduced allocation of substrate to biomass formation and increased relative contribution of maintenance‐energy requirements. The strongly increased m_s_ at low pH is clearly disadvantageous for industrial scale production of dicarboxylic acids and, moreover, is likely to be further enhanced in the presence of high product concentrations. For example, high concentrations of organic acids have been shown to cause increased maintenance‐energy requirements at low pH (Abbott et al., 2007; Abbott, Suir, Van Maris, & Pronk, 2008). From an economic perspective, the physiological impacts of low pH on *S. cerevisiae* constitute a trade‐off between fermentation costs and costs for downstream processing. The complexity of the observed physiological and transcriptional responses indicates that improving robustness under industrial conditions is unlikely to be achieved by individual genetic modifications. Instead, exploration of yeast biodiversity (Palma, Guerreiro, & Sá‐Correia, 2018), evolutionary engineering (Mans, Daran, & Pronk, 2018) and/or genome‐shuffling approaches (Magocha et al., 2018; Steensels, Gorkovskiy, & Verstrepen, 2018) may offer interesting possibilities.

## Supporting information

Supplementary information.fv

Supplementary information

Supplementary information

Supplementary information

Supplementary information
