# Supplementary material for: Physiological responses of Saccharomyces cerevisiae to industrially relevant conditions: Slow growth, low pH, and high CO2 levels
Source: Biotechnol Bioeng. 2020 Jan 22;117(3):721–35. doi: 10.1002/bit.27210 (PMC7028085; doi:10.1002/bit.27210)
Supplement: Supplementary file 1 — Supplementary information.fv [file BIT-117-721-s001.docx]

# Appendix1: Materials & Methods

#### **Appendix 1.1 Bioreactor operations of aerobic, carbon-limited cultures**

For each bioreactor experiment a single freezer stock was inoculated to 100 mL Synthetic Medium with 2% glucose (Verduyn et al. 1992) in 500 mL shake flasks and incubated over night at 30 °C in an Innova 44 incubator (New Brunswick Scientific, Edison, NJ, USA) set at 200 rpm. Cells growing exponentially in fresh medium were used to inoculate the bioreactors at an initial OD of 0.2 – 0.4, measured at 660 nm in a Libra S12 spectrophotometer (Biochrom, Cambridge, UK).

The bioreactor was stirred at 800 rpm and the temperature was kept constant at 30 °C. The medium flow rate was set by a calibrated inflow pump, while the culture volume in the bioreactor was kept constant by the effluent pump controlled by an electrical level sensor. An Applisens pH probe (Applikon, Delft, The Netherlands) was used to monitor the pH, which was maintained at either pH 5.0 or pH 3.0 by the automatic addition of a 12.5% NH_4_OH solution, prepared by diluting a 25% ammonia solution in a 1:1 ratio with demineralized water which was then filter sterilized (0.22 μm pore size SFCA bottle-top filter; Thermo Scientific, Waltham, MA).

The operation of retentostats was essentially performed as described before (Vos et al. 2016). Upon reaching steady state (see below), the retentostat phase was initiated by switching from a regular effluent port to an effluent port equipped with an Applisense filter assembly (Applikon, Delft, The Netherlands) with a 0.2 μm pore sized filter that was inserted in the reactor prior to autoclaving. Prior to autoclaving the filter was mounted on the filter support and wetted by overnight incubation in 96% ethanol and subsequently rinsed with a phosphate buffer saline solution.

The gas flow rate of the inflowing gas was set to 700 mL/min (0.5 vvm) and the composition was either compressed air (0.04% CO_2_) or an in-line mix of compressed air and pure CO_2_ (>99.7% purity, Linde Gas Benelux, Schiedam, The Netherlands) at a ratio of 1:1 to obtain 50% concentration of CO_2_ in the inflowing gas by means of two mass flow controllers (Brooks, Hatfield, PA, USA). The exhaust gas from these chemostat and retentostat cultures was cooled with a condenser at 2 °C and dried with a PermaPure Dryer (model MD 110-8P-4; Inacom Instruments, Veenendaal, The Netherlands). The dried gas was analyzed for carbon dioxide and oxygen concentration with a combined paramagnetic/infrared off-gas analyzer NGA2000 Analyser (Rosemount, Baar, Switserland).

The medium composition for these cultivations is based on the medium described previously (Verduyn et al. 1992) and was modified to sustain higher biomass concentrations by increasing the vitamin concentration (2-fold) and the trace element solution (1.5-fold) (adaptations previously described by (Vos et al. 2016)) with 20 g/L as growth limiting nutrient and increased concentrations of iron sulfate in EDTA and biotin as described by Royal DSM, The Netherlands (Jansen et al. 2014) , final medium concentrations 7.5 mg/L and 1.1 mg/L respectively. The glucose concentration for both chemostat as well as retentostat phases was 20 g/L. For cultivations at pH 3, the medium for continuous cultivations was set to pH 3.0 by addition of 18N H_2_SO_4_ (Sigma Aldrich, Zwijndrecht, The Netherlands).

#### **Appendix1.2 Bioreactor operations of aerobic, nitrogen-limited cultures**

A bench scale 7 liters bioreactor (Applikon, Delft, The Netherlands) with a working volume of 5 liters was incubated with grown pre-cultures and firstly ran in batch mode. After the nitrogen source was depleted in the batch culture, the bioreactor was switched to chemostat cultivation mode at a dilution rate of 0.025 h^-1^. Retentostat cultivation was performed at the same dilution rate after steady state condition was achieved during the preceding N-limited chemostat phase.

From the start of the retentostat phases the glucose and nitrogen concentrations in the medium added to the bioreactor were gradually decreased by the control of two feed pumps, drawing medium from two different medium vessels. This decrease resulted in a similar decrease in limiting nutrient concentration as described before (Liu et al. 2019; Vos et al. 2016).

The bioreactor culture temperature was kept at 30 and stirring was maintained at 500 rpm. Fully aerobic condition was achieved by sparging 0.5 vvm compressed air controlled by a mass flow controller (Brooks 5850 TR, Hatfield, PA, USA). Culture pH was controlled at 3 ± 0.05 by adding either 2 M KOH or 2M H_2_SO_4_ using a Biostat Bplus controller (Sartorius BBI Systems, Melsungen, Germany).

The media compositions of preculture, batch, chemostat and retentostat referred to Liu (Liu et al. 2019).

#### **Appendix1.4 Extracellular metabolites**

Extracellular metabolites of the cultures under carbon limited conditions were obtained by centrifuging culture samples (3 min at 13000 rpm) of which the supernatant was analyzed based on high-performance liquid chromatography (HPLC) analysis on an Agilent 1100 HPLC (Agilent Technologies, Santa Clara, CA, USA) equipped with an Aminex HPX-87H ion-exchange column (BioRad, Veenendaal, The Netherlands) kept at 60 ºC, eluted with sulfuric acid (5 mM, 0.6 mL/min). Detection of glucose, ethanol and glycerol was performed by a refractive-index detector (Agilent G1362A), which were not detected above 1mM in the carbon-limited cultures. Detection of acetate, succinic acid, lactic acid and pyruvic acid was a dual-wavelength absorbance detector (Agilent G1314A; 214 nm) and were typically below 0.8 mM under carbon limitation. Residual glucose in these cultures was determined as described by Mashego et al. based on quenching of culture broth by cold steel beads (Mashego et al. 2003) and direct filtering with a 0.45 μm PVDF membrane (Millex HV, Merck, Cork, Ireland). Residual glucose concentrations were determined based on enzymatic determination kit (D-glucose HK Assay Kit, Megazyme, Wicklow, Ireland) in a 96-well plate. Absorbance at 340 nm was analyzed on a GENios Pro absorbance plate reader (Tecan, Männedorf, Switzerland).

Effluent samples of the nitrogen limited cultures at low pH were harvested from the sample port connected to the installed 0.22 µm retentostat filters. Glucose, ethanol, glycerol, succinate, lactate, and acetate concentrations in the culture were quantified by HPLC using a Bio-Rad HPX-87H. The column was eluted with phosphoric acid (1.5 mM, 0.6 mL/min). The detection of glucose, ethanol and glycerol concentrations were performed by a refractive-index detector (Walters 2414) and the succinate, lactate, acetate concentrations with a UV detector (Walters 484; 210 nm). Extracellular amino acids were analyzed by GC-MS as describe by de Jonge et al. (de Jonge et al. 2011). The residual ammonium concentration was determined by an ammonium cuvette test (0.02 - 2.5 mg/L NH_4_^+^) (Hach Lange GMBH, Dusseldorf, Germany).

#### **Appendix1.5 Viability determination from FACS**

Viability measurements using fluorescence-assisted cell sorting (FACS) was performed essentially as described before (Brickwedde et al. 2018). Briefly, cultures were analyzed on a BD FACSAria™ II SORP Cell Sorter (BD Biosciences, Franklin Lakes, NJ) equipped with 355, 445, 488, 561, and 640 nm lasers and a 70 μm nozzle, and operated with filtered FACSFlow™ (BD Biosciences). Correct cytometer performance was evaluated prior to each experiment by running a CST cycle with corresponding CS&T Beads (BD Biosciences). Drop delay for sorting was determined by running an Auto Drop Delay cycle with Accudrop Beads (BD Biosciences). Morphology of the cells was analyzed by plotting forward scatter (FSC) against side scatter (SSC). Ninety-Six single cells were sorted onto 96-well format Nunc omnitray (Thermo Scientific) plates containing YPD agar using a “single cell” sorting mask, corresponding to a yield mask of 0, a purity mask of 32 and a phase mask of 16. Viability was measured as the average percentage of sorted cells able to form a colony after 48 h incubation at 30°C on three triplicate plates.

#### **Appendix 1.6 Transcriptome analysis**

Samples for transcriptome analysis were taken from carbon-limited chemostat and retentostat cultures and was performed by quenching culture broth directly into liquid nitrogen to immediately stop mRNA turnover (Mendes et al. 2013; Piper et al. 2002). Samples were stored immediately at -80 °C. For long term storage, cells were broken and RNA stabilized by the AE-phenol chloroform method (Schmitt et al. 1990). Total RNA extraction was performed as described previously (Mendes et al. 2013). The quality of the total extracted RNA was evaluated with an Agilent 2200 Tapestation (Agilent Technologies, Santa Clara, CA) and the RNA concentration was determined using a Qubit 2.0 Fluorometer (Thermo Fisher Scientific) combined with the Qubit RNA BR assay Kit (Thermo Fisher Scientific).

Library preparation and RNA sequencing of the carbon-limited cultures in this study were performed by Novogene Bioinformatics Technology Co., Ltd. (Yuen Long, Hong Kong). Sequencing was done with Illumina paired end 150 bp sequencing read system (PE150) using a 250∼300 bp insert strand specific library which was prepared by Novogene. For the library preparation, mRNA enrichment was done using oligo(dT) beads. After random fragmentation of the mRNA, cDNA was synthetized from the mRNA using random hexamer primers. Afterward, second strand synthesis was done by addition of a custom second strand synthesis buffer (Illumina), dNTPs, RNase H and DNA polymerase I. Finally, after terminal repair, A ligation and adaptor ligation, the double stranded cDNA library was finalized by size selection and PCR enrichment. The sequencing data for these samples obtained by Novogene on a HiSeq2500 had an average read depth of 14 million reads. For each sample, reads were aligned to the reference genome (S288C; (Engel et al. 2014)) with the two-pass STAR procedure (Dobin et al. 2013). In the first pass, we assembled a splice junction database which was used to inform the second round of alignments. Introns were allowed to be between 15 and 4000 bp, and soft clipping was disabled to prevent low quality reads from being spuriously aligned. Ambiguously mapped reads were removed. Expression was quantified per transcript using ht-seq count in union intersection mode (Anders et al. 2015). To exclude lowly expressed genes from the analysis, genes with an average FPKM value for all samples below 10 were removed. Subsequently raw counts were normalized by TMM normalization using the EdgeR package (Robinson et al. 2009) and differentially expressed genes were determined with an absolute fold change (FC) > 2 and a False Discovery Rate (FDR) < 0.005. When differentially expressed genes were determined compared to pH 5, 0.04% CO_2_ conditions, a subset of 16 genes that was differentially expressed in all three conditions, showing enrichment for genes in the pheromone response (Appendix) was further excluded for further gene set enrichment analysis. Gene-set enrichment analysis based on hypergeometric distribution analysis using the Piano package was performed with a Benjamini-Hochberg corrected p-value < 0.05 for GO-terms (obtained from Ensembl on 07-02-2019), Transcription factor sets (Harbison et al. 2004) and a set of personal categories for organic acids (Abbott et al. 2007), ageing (Aragon et al. 2008), stationary phase (Martinez et al. 2004), autophagy (Reggiori and Klionsky 2013), stress response (Eastmond and Nelson 2006; Gasch et al. 2000; Gibney et al. 2013), growth rate responsive genes (Brauer et al. 2008; Castrillo et al. 2007; Regenberg et al. 2006; Vos et al. 2016), cell cycle genes (Cho et al. 1998; Spellman et al. 1998), low pH (Abbott et al. 2008; Chen et al. 2009; de Lucena et al. 2015a; de Lucena et al. 2015b; Kapteyn et al. 2001; Kawahata et al. 2006), CO_2_ (Aguilera et al. 2005) and genes involved in central carbon metabolism. Trend analysis of per gene mean-normalized expression versus the growth rate in retentostats was performed using the maSigPro R-package (Conesa et al. 2006; Nueda et al. 2014). In this package first the genes with a trend significantly different from the mean are selected with a Benjamini-Hochberg corrected p < 0.03 and an R^2^ > 0.8. Subsequently the regression parameters for two clusters were identified with a significance cutoff of 0.05.

For comparison of transcriptome data generated in the present study to data of previous aerobic retentostat cultures (Vos et al. 2016), total RNA was extracted (see above) and sequencing was done with Illumina single end 150 bp sequencing read system (PE150) using a 100-400 bp insert strand specific library which was prepared by Baseclear. For the library preparation, mRNA enrichment was done using oligo(dT) beads. The Illumina Truseq stranded RNA-seq library preparation kit was used. The sequencing data for these samples obtained by Baseclear had at least a sequencing depth of 21.5 million reads. Mapping of these reads was performed as described above.

#### **Appendix 1.7 Trehalose and glycogen quantification**

Analysis of trehalose was performed directly by HPLC as described above. Analysis of glycogen was done by analysis of glucose released from glycogen by enzymatic determination after overnight incubation at 57 ºC of samples with α-amyloglucosidase from *Aspergillus niger* (Sigma-Aldrich, Zwijndrecht, The Netherlands). The enzymatic determination of glucose was performed as described above for residual glucose determination.

Abbott DA, Knijnenburg TA, De Poorter LMI, Reinders MJT, Pronk JT, Van Maris AJA. 2007. Generic and specific transcriptional responses to different weak organic acids in anaerobic chemostat cultures of *Saccharomyces cerevisiae*. FEMS Yeast Research 7(6):819-833.

Abbott DA, Suir E, Van Maris AJA, Pronk JT. 2008. Physiological and transcriptional responses to high concentrations of lactic acid in anaerobic chemostat cultures of *Saccharomyces cerevisiae*. Applied and Environmental Microbiology 74:5759-68.

Aguilera J, Petit T, De Winde JH, Pronk JT. 2005. Physiological and genome-wide transcriptional responses of *Saccharomyces cerevisiae* to high carbon dioxide concentrations. FEMS Yeast Research 5(6-7):579-593.

Anders S, Pyl PT, Huber W. 2015. Genome analysis HTSeq — a Python framework to work with high-throughput sequencing data. Bioinformatics 31(2):166-169.

Aragon AD, Rodriguez AL, Meirelles O, Roy S, Davidson GS, Tapia PH, Allen C, Joe R, Benn D, Werner-Washburne M. 2008. Characterization of differentiated quiescent and nonquiescent cells in yeast stationary-phase cultures. Mol Biol Cell 19(3):1271-80.

Brauer MJ, Huttenhower C, Airoldi EM, Rosenstein R, Matese JC, Gresham D, Boer VM, Troyanskaya OG, Botstein D. 2008. Coordination of growth rate, cell cycle, stress response, and metabolic activity in yeast. Molecular biology of the cell 19(1):352-67.

Brickwedde A, Brouwers N, Broek MVD, Gallego Murillo JS, Fraiture JL, Pronk JT, Daran J-MG. 2018. Structural, physiological and regulatory analysis of maltose transporter genes in *Saccharomyces eubayanus* CBS 12357T. frontiers in Microbiology 9:1-18.

Castrillo JI, Zeef LA, Hoyle DC, Zhang N, Hayes A, Gardner DCJ, Cornell MJ, Petty J, Hakes L, Wardleworth R, B. and others. 2007. Growth control of the eukaryote cell- a systems biology study in yeast. Journal of biology 6(4).

Chen AKL, Gelling C, Rogers PL, Dawes IW, Rosche B. 2009. Response of *Saccharomyces cerevisiae* to stress-free acidification. Journal of Microbiology 47:1-8.

Cho RJ, Campbell MJ, Winzeler EA, Steinmetz L, Conway A, Wodicka L, Wolfsberg TG, Gabrielian AE, Landsman D, Lockhart DJ and others. 1998. A genome-wide transcriptional analysis of the mitotic cell cycle. Molecular Cell 2:65-73.

Conesa A, Nueda MJ, Ferrer A, Talon M. 2006. maSigPro: a method to identify significantly differential expression profiles in time-course microarray experiments. Bioinformatics 22(9):1096-102.

de Jonge LP, Buijs NA, ten Pierick A, Deshmukh A, Zhao Z, Kiel JA, Heijnen JJ, van Gulik WM. 2011. Scale-down of penicillin production in *Penicillium chrysogenum*. Biotechnol J 6(8):944-58.

de Lucena RM, Elsztein C, de Barros Pita W, de Souza RB, de Sá Leitão Paiva Júnior S, de Morais Junior MA. 2015a. Transcriptomic response of *Saccharomyces cerevisiae* for its adaptation to sulphuric acid-induced stress. Antonie van Leeuwenhoek, International Journal of General and Molecular Microbiology 108:1147-1160.

de Lucena RM, Elsztein C, de Barros Pita W, de Souza RB, de Sá Leitão Paiva Júnior S, de Morais Junior MA. 2015b. Transcriptomic response of *Saccharomyces cerevisiae* for its adaptation to sulphuric acid-induced stress. Antonie van Leeuwenhoek, International Journal of General and Molecular Microbiology 108(5):1147-1160.

Dobin A, Davis CA, Schlesinger F, Drenkow J, Zaleski C, Jha S, Batut P, Chaisson M, Gingeras TR. 2013. STAR: ultrafast universal RNA-seq aligner. Bioinformatics 29(1):15-21.

Eastmond DL, Nelson HC. 2006. Genome-wide analysis reveals new roles for the activation domains of the Saccharomyces cerevisiae heat shock transcription factor (Hsf1) during the transient heat shock response. J Biol Chem 281(43):32909-21.

Engel SR, Dietrich FS, Fisk DG, Binkley G, Balakrishnan R, Costanzo MD, Dwight SS, Hitz BC, Karra K, Nash RS and others. 2014. The Reference Genome Sequence of *Saccharomyces cerevisiae* : Then and Now. G3 4(3):389-398.

Gasch AP, P.T. S, Kao CM, Carmel-Harel O, Eisen MB, Storz G, Botstein D, Brown PO. 2000. Genomic Expression Programs in the Response of Yeast Cells to Environmental Changes. molecular Biology of the Cell 11:4241-4257.

Gibney PA, Lu C, Caudy AA, Hess DC, Botstein D. 2013. Yeast metabolic and signaling genes are required for heat-shock survival and have little overlap with the heat-induced genes. Proc Natl Acad Sci U S A 110(46):E4393-402.

Harbison CT, Gordon DB, Lee TL, Rinaldi NJ, Macisaa KD, Danford TW, Hannett NM, Tagne J-B, Reynolds DB, Yoo J and others. 2004. Transcriptional regulatory code of a eukaryotic genome. Nature 431:99-104.

Jansen MLA, Heijnen JJ, Verwaal R; DSM II Assets B.V., assignee. 2014 20-11-2014. Process for preparing dicarboxylic acids employing fungal cells. United States.

Kapteyn JC, ter Riet B, Vink E, Blad S, De Nobel H, Van Den Ende H, Klis FM. 2001. Low external pH induces HOG1-dependent changes in the organization of the *Saccharomyces cerevisiae* cell wall. Molecular Microbiology 39:469-480.

Kawahata M, Masaki K, Fujii T, Iefuji H. 2006. Yeast genes involved in response to lactic acid and acetic acid: acidic conditions caused by the organic acids in *Saccharomyces cerevisiae* cultures induce expression of intracellular metal metabolism genes regulated by Aft1p. FEMS yeast research 6:924-36.

Liu Y, el Bouhaddani A, Pronk JT, van Gulik WM. 2019. Quantitative physiology of non-energy-limited retentostat cultures of *Saccharomyces cerevisiae* at near-zero specific growth rates. BioRxiv.

Martinez MJ, Roy S, Archuletta AB, Wentzell PD, Anna-Arriola SS, Rodriguez AL, Aragon AD, Quinones GA, Allen C, Werner-Washburne M. 2004. Genomic analysis of stationary-phase and exit in Saccharomyces cerevisiae: gene expression and identification of novel essential genes. Mol Biol Cell 15(12):5295-305.

Mashego MR, van Gulik WM, Vinke JL, Heijnen JJ. 2003. Critical evaluation of sampling techniques for residual glucose determination in carbon-limited chemostat culture of *Saccharomyces cerevisiae*. Biotechnol Bioeng 83(4):395-9.

Mendes F, Sieuwerts S, de Hulster E, Almering MJH, Luttik MAH, Pronk JT, Smid EJ, Bron PA, Daran-Lapujadea P. 2013. Transcriptome-based characterization of interactions between *Saccharomyces cerevisiae* and *Lactobacillus delbrueckii* subsp. bulgaricus in lactose-grown chemostat cocultures. Applied and Environmental Microbiology 79(19):5949-5961.

Nueda MJ, Tarazona S, Conesa A. 2014. Next maSigPro: Updating maSigPro bioconductor package for RNA-seq time series. Bioinformatics 30(18):2598-2602.

Piper MDW, Daran-Lapujade P, Bro C, Regenberg B, Knudsen S, Nielsen J, Pronk JT. 2002. Reproducibility of Oligonucleotide Microarray Transcriptome Analyses. Journal of Biological Chemistry 277(40):37001-37008.

Regenberg B, Grotkjaer T, Winther O, Fausboll A, Akesson M, Bro C, Hansen LK, Brunak S, Nielsen J. 2006. Growth-rate regulated genes have profound impact on interpretation of transcriptome profiling in Saccharomyces cerevisiae. Genome Biol 7(11):R107.

Reggiori F, Klionsky DJ. 2013. Autophagic processes in yeast: mechanism, machinery and regulation. Genetics 194(2):341-61.

Robinson MD, McCarthy DJ, Smyth GK. 2009. edgeR: A Bioconductor package for differential expression analysis of digital gene expression data. Bioinformatics 26(1):139-140.

Schmitt ME, Brown TA, Trumpower BL. 1990. A rapid and simple method for preparation of RNA from *Saccharomyces cerevisiae*. Nucleic Acids Research 18(10):3091-3092.

Spellman PT, Sherlock G, Zhang MQ, Iyer VR, Anders K, Eisen MB, Brown PO, Botstein D, Flutcher B. 1998. Comprehensive identification of cell-cycle-regulated genes of the yeast *Saccharomyces cerevisiae* by microarray hybridization. molecular Biology of the Cell 9:3273-3297.

Verduyn C, Postma E, Scheffers WA, Van Dijken JP. 1992. Effect of benzoic acid on metabolic fluxes in yeasts: a continuous-culture study on the regulation of respiration and alcoholic fermentation. Yeast (Chichester, England) 8:501-17.

Vos T, Hakkaart XDV, de Hulster EAF, van Maris AJA, Pronk JT, Daran-Lapujade P. 2016. Maintenance-energy requirements and robustness of *Saccharomyces cerevisiae* at aerobic near-zero specific growth rates. Microbial Cell Factories 15(1):111-111.
