# Supplementary material for: Physiological responses of Saccharomyces cerevisiae to industrially relevant conditions: Slow growth, low pH, and high CO2 levels
Source: Biotechnol Bioeng. 2020 Jan 22;117(3):721–35. doi: 10.1002/bit.27210 (PMC7028085; doi:10.1002/bit.27210)
Supplement: Supplementary file 2 — Supplementary information [file BIT-117-721-s002.docx]

Appendix 2 : Supplementary figures

## Supplementary figure 1


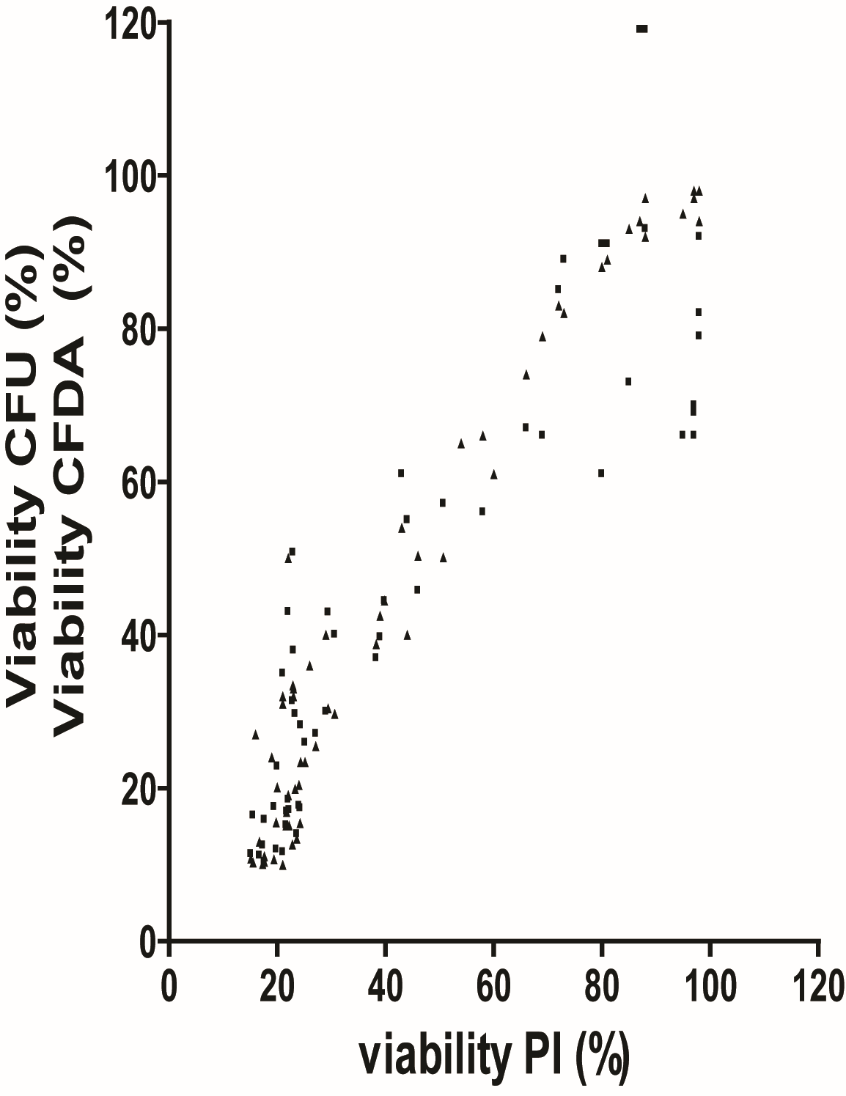


Correlation plot for viability based on Colony Forming Units (CFU; squares) and 5-CFDA, AM (CFDA, triangles) versus viability based on Propidium Iodide (PI) staining for all experiments in this study

## Supplementary figure 2


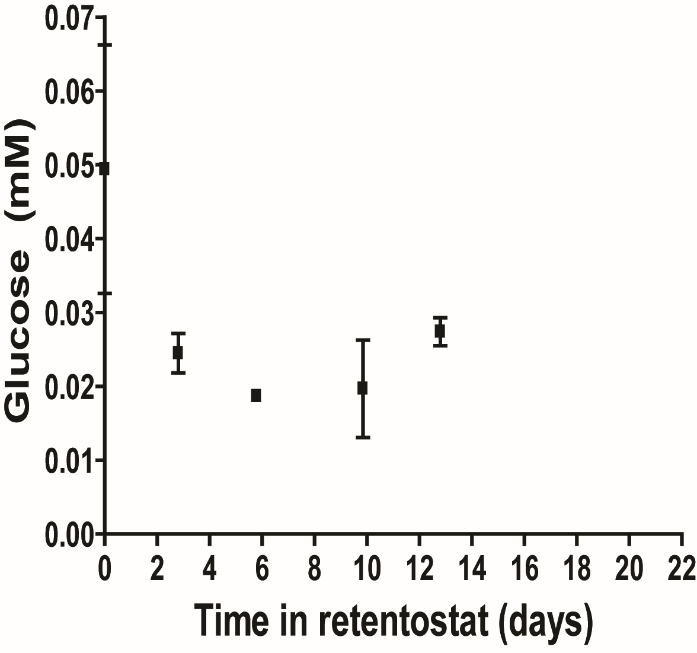


Residual glucose concentration in carbon-limited retentostat experiments at “industrially relevant” conditions (pH3, 50% CO_2_). Error bars indicate the Mean average deviation from duplicate experiments.

## Supplementary figure
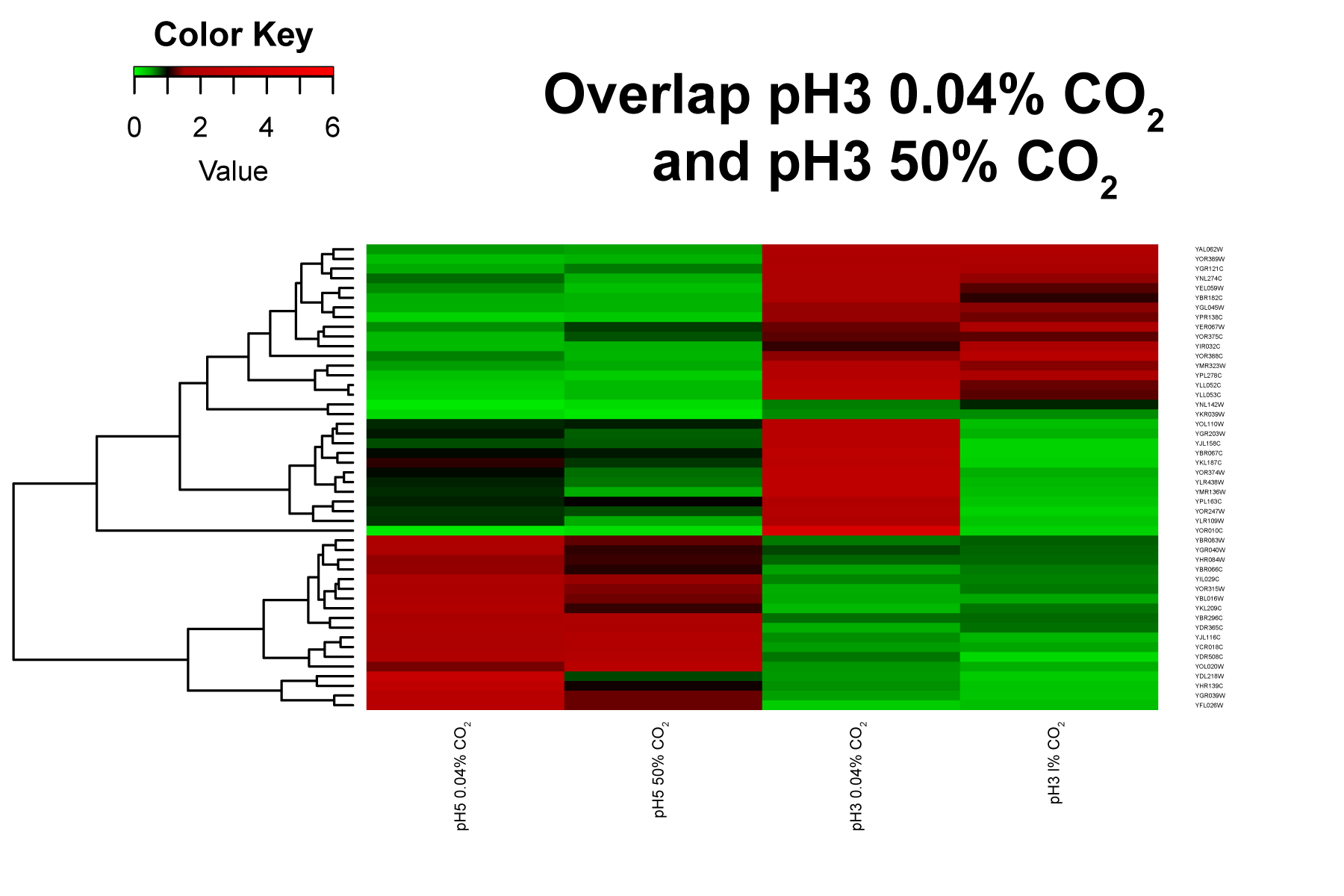
3

Heat map for the 48 genes in section δ (Figure 5) that are differentially expressed in both low pH3 combined with 50% CO_2_ conditions, as well as with pH3 alone.
